# Changes in the Seminiferous Epithelium of the Testes during Postnatal Development in Assam Goat

**DOI:** 10.1155/2012/620924

**Published:** 2012-02-14

**Authors:** Kamal Sarma, J. Devi

**Affiliations:** Faculty of Veterinary Sciences & Animal Husbandry, Sher-e-Kashmir University of Agricultural Sciences and Technology of Jammu, R.S. Pura, 181 102, India

## Abstract

The present work is conducted to elucidate the postnatal development of the seminiferous epithelium of the testes of the Assam goats from 0 day to 10 months of age. A total of eighteen Assam goats divided into six age groups, namely, group-I (0-day), group-II (2 months), group-III (4 months), group-IV (6 months), group-V (8 months), and group-VI (10 months), consisting of 3 animals in each group were used in this study. The seminiferous tubules did not have lumina up to the age of 2 months, hence called the sex cords, and these contained centrally placed gonocytes and peripherally located sustentacular cells. Initiation of spermatogenesis started in 4-month old kids. Luminization process was completed by 6 months of age with all the seminiferous tubuyes having well-developed lumina at this age. These seminiferous tubules contained all the spermatogenic cells of the adult testis. Onset of puberty was observed to be established at 6 months of age in the Assam goats as evidenced by presence of spermatozoa adhering to the adluminal border of the Sertoli cells as well as in the tubular lumen. The histomorphology of various cells of the seminiferous epithelium has been described.

## 1. Introduction

India possesses 122.92 millions of goats of which 29.06 lacs are found in Assam [1]. Goat rearing has tremendous potential in the northeastern states particularly among the small and marginal farmers and landless laborers because of very low initial investment and adequate financial returns. More than 85 percent of the population in this region is nonvegetarian, and chevon is preferred by all as it has no religious taboo.

Postnatal anatomical studies on the male genital system at various ages, particularly the testis and its tubular system, are important to know the anatomical growth and development. A few anatomical studies on testes are reported in goats, *namely*, biometry of the testes in the Sirohi goats [2], testicular measurements in the Assam local X Beetal goats [3], and testicular growth in the British Saanen, Alpine, and Toggenburg breeds of bucks [4]. Some works were also conducted in other ruminants elucidating morphology and biometry of the testes such as buffalo [5] and rams [6].

 This study is the first report for the postnatal development of the seminiferous epithelium in the Assam goat.

## 2. Materials and Methods

A total of 18 male Assam goats varying in age from 0-day to 10 months were used in the present study. The animals were divided into six age groups, *namely *group-I (0-day), group-II (2 months), group-III (4 months), group-IV (6 months), group-V (8 months), and group-VI (10 months) consisting of three animals in each group. The age of the goats were estimated from birth records. Each animal was weighed using Spring Balance to record the body weight. The animals were sedated by intramuscular injection of Siquil @ 1 mg/Kg body weight and subsequently anaesthetized by intravenous injection of Intravel Sodium @ 15 mg/Kg body weight [7]. After induction of proper level of anesthesia, the animals were sacrificed.

Tissue pieces were collected from three different regions of the testis, *namely*, dorsal (part attached to the caput epididymis), middle, and ventral (part attached to the cauda epididymis) and subsequently fixed in Bouin's solution [8]. All the tissues were processed for paraffin sections [8] by alcohol-xylene method using cedar wood oil. Sections were cut at 5 *μ* thickness using a Rotary Microtome (Thermo, Germany) and stained for various stains, *namely,* haematoxylin and eosin, Masson's trichrome stain for collagen, Mallory's method for collagen, Weigert's method for elastic fibres, Gomori's method for reticular fibres, Berg's method for spermatozoa [8], PAS-haematoxylin method for nuclear staining, and Lillie's allochrome method for basement membrane [9].

## 3. Results and Discussion

### 3.1. Seminiferous Tubules

**Figure 1:**
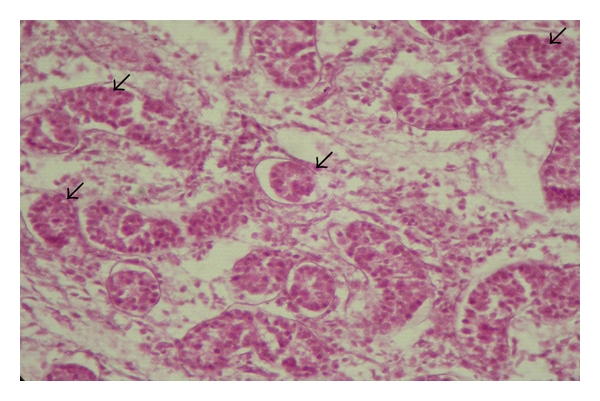
Photomicrograph of the testis of a day-old kid showing the sex cords (arrows). H&E, 400 X.

**Figure 2:**
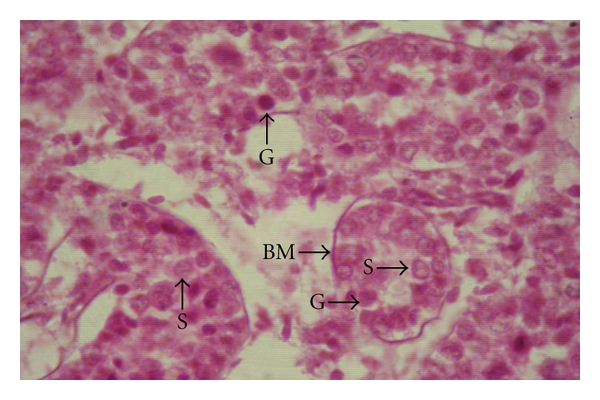
Photomicrograph showing the gonocytes (G), sustentacular or Sertoli cells (S) and the basement membrane (BM) of the sex cords in the testis of a day-old male kid H&E, 1000 X.

**Figure 3:**
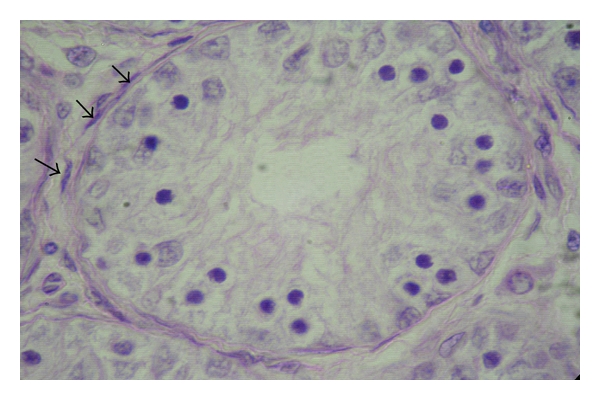
Photomicrograph showing the peritubular cells (arrows) surrounding the seminiferous tubules in the testis of a two-month-old male kid, H&E, 400 X.

 In the animals of groups I and II, no lumen was found in the seminiferous tubules; hence, these were called the sex cords (Figure 1). These solid sex cords contained peripheral primitive Sertoli cells and large centrally placed germ cells located in an eosinophilic material (Figure 2). The basement membranes of the sex cords were surrounded by very fine reticular and collagenous fibres. The gonocytes were large cells with spherical nucleus of homogenous nucleoplasm. They showed variable degree of degeneration in the form of vacuolated cytoplasm and different stages of karyolysis. The primitive Sertoli cells were polygonal or cone-shaped cells with lighter and relatively homogenous cytoplasm. Nuclei of these primitive Sertoli cells showed heterochromatin material within foldings in the nucleolemma. Nucleoli were not distinct. At this stage, these cells were more in number than the gonocytes. Distinct basement membrane was seen around the sex cords. The sex cords were convoluted and surrounded by one or more layers of peritubular cells in the animals of the first two age groups (Figure 3). Similar observations were made by Singh [10] in buffalo testis. An increase in tubular convolution associated with decreased intertubular space was observed with the advancement of age in the Assam goats. This sort of interrelationship between the tubular and interstitial compartments represented a delicately balanced complex biological unit [11].

**Figure 4:**
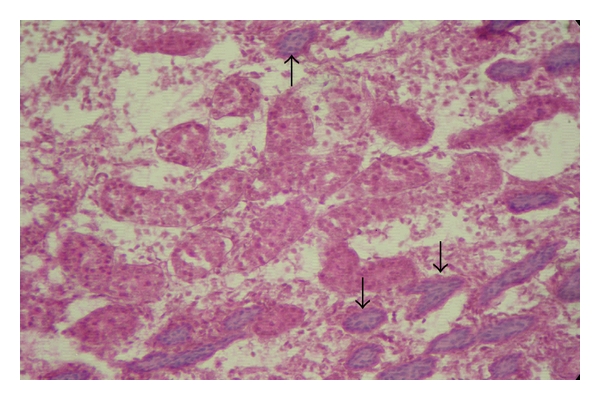
Photomicrograph of the testis of a day-old kid showing the presence of sex-cord-like structures (arrows) filled with basophilic material, H&E, 100 X.

 In addition to these sex cords having primitive germ cells (gonocytes) and sustentacular cells, certain sex-cord-like structures were seen in the testes of day-old kids (group-I). These sex-cord-like structures were distributed just below the testicular capsule and filled with light basophilic material (Figure 4), which might be the developing sex cords. However, no such reports were found available in the literature to compare to the present findings.

**Figure 5:**
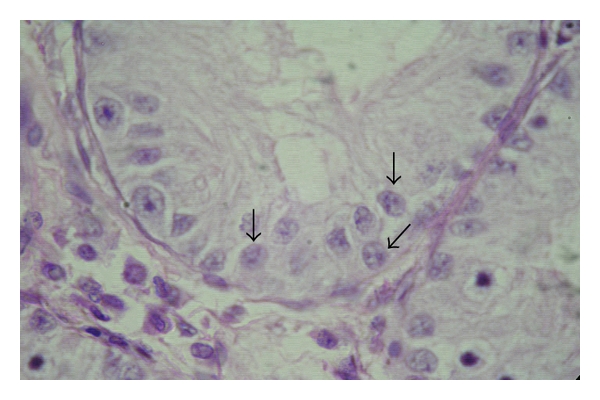
Photomicrograph of the testis of a two-month old male kid showing the presence of propermatogonia (arrows), H&E., 1000 X.

 With progression of age, these gonocytes started migrating towards the basement membrane of the sex cords. These cells were termed as prespermatogonia which could be seen in the sex cords of 2-month-old kids (group-II) (Figure 5). Such centrifugal movements of the gonocytes to form prespermatogonia were also observed earlier by Singh [10] in buffalo calves and [31] in domestic animals. Further, Lee and Burger [12] also described that the central gonocytes migrated towards the periphery of the sex cords with advancement of age and entered three proliferative phases by a series of mitotic divisions, and prespermatogonia were formed which gave rise to the future generations of spermatogonia. In Japanese Black bulls, the gonocytes were replaced by prespermatogonia at the age of 3 months [13]. Prespermatogonia differed from spermatogonia in terms of their uniform spherical contour and looser chromatin distribution.

Luminization of majority of these cords initiated at 2 months of age (Group-II), and completed by 6 month of age (group-VI) in the Assam goats. Similar observations were also reported in ram [14]. The age of luminization of these sex cords was reported to be variable in domestic animals being at 20–24 weeks of postnatal age in cattle [15], 90 days in rams [16], and 3 months of age in buffaloes [17]. Most of the seminiferous tubules in 4-month-old kids (group-III) had distinct lumen. The process of luminization of the seminiferous tubules was characterized by disintegration of the central cells and liquefaction of the intercellular substance of the sex cords. However, few cords with incomplete lumina were noticed in 4-month-old kids (group-III). Goyal and Dhingra [18] reported that the stratification of seminiferous tubular epithelium was seen at 50–52 weeks of age in buffalo. Increased tubular convolution with advancing age of the male kids as recorded in this study was also reported earlier in goats [19].

**Figure 6:**
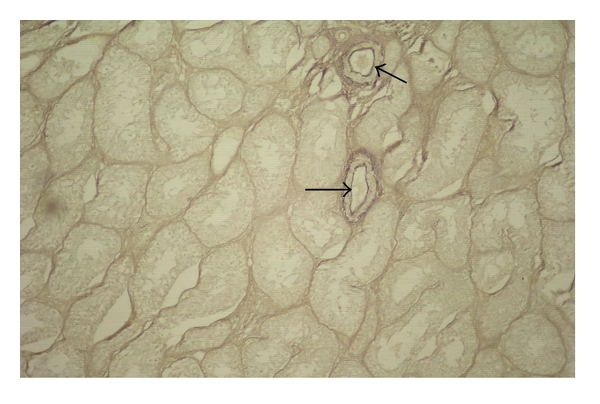
Photomicrograph of the testis of a four-month-old male kid showing presence of elastic fibres (arrows) around blood vessels, Hart's method, 100 X.

 The seminiferous tubules had prominent basement membranes supporting the germinal epithelium. A distinct network of reticular fibres interspersed with fine collagenous fibres were marked in the basement membrane of the tubules in the male goats of all the age groups. Elastic fibres were often found around the blood vessels and in the internal elastic membrane (Figure 6). Increase in tubular convolution and decrease of intertubular space was marked as the age of the animals advanced.

 The seminiferous tubules were lined by stratified epithelium in kids from 4 months of age (group-III) onwards comprising type A and B spermatogonia, Sertoli cells, and primary spermatocytes. The process of spermatogenesis was seen to be started in 4-month-old kids (group-III) as spermatogonia were first seen in the seminiferous epithelium at this age (120 days). All the seminiferous tubules in the bucks from 6 months of age (group-IV) onwards had distinct lumina. The stratified epithelium of these seminiferous tubules contained type A and B spermatogonia; leptotene, zygotene, pachytene, and diplotene primary spermatocytes; secondary spermatocytes; round spermatids; elongated spermatids and spermatozoa. The intermediate type of spermatogonia could not be found in any of the animals in this study. The spermatogonia were located close to the basement membranes, while their successive descendants were progressively closer to the lumina of the seminiferous tubules.

### 3.2. The Spermatogenic Cells

**Figure 7:**
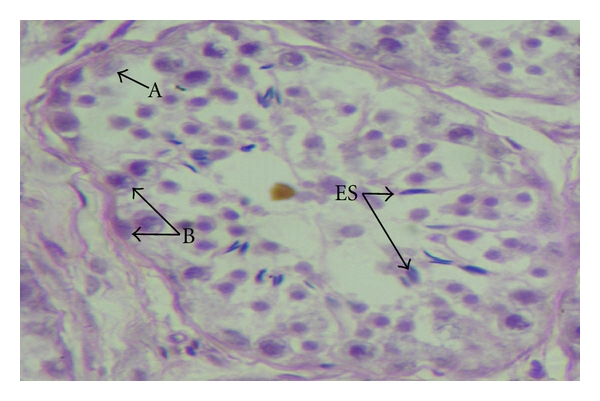
Photomicrograph showing type-A spermatogonia (A), type-B spermatogonia (B), and elongated spermatids (ES) in the seminiferous epithelium of the testis of an eight-month-old buck, H&E., 400 X.

Based on the distribution pattern of chromatin and size of the spermatogonia, type-A and type-B spermatogonia were distinguished in this study. Similar types of spermatogonia were also reported in rat [20], Dwarf Nigerian ram [21], stallion [22], bulls [23], and goat [24]. The intermediate type of spermatogonia was not classified in the present study. The criteria of these cells having irregular distribution of numerous Fulgent-positive chromatin granules in ovoid nucleus and their attachment to the nucleoli and nuclear membrane did not hold good in goat. Similar observations were also made by Bordoloi and Dhingra [24]. The type A spermatogonia (Figure 7) were large oval or elliptical-shaped cells located close to the basement membranes of the seminiferous tubules. They had large and oval nuclei containing uniform chromatin material. The nucleoli were located at random within the nucleus, and their numbers were usually more than one. The cytoplasmic periphery of the cell was weakly eosinophilic, and the long axis of the cell was placed parallel to the basal lamina.

 The type B spermatogonia were proportionately rounded cells with distinct spherical nuclei. The chromatin substance was observed as distinct clumps which usually adhere to the nuclear membrane. The nucleoli were located at the centre of the nucleus, but deviations were also noted. The cytoplasm was lightly eosinophilic (Figure 7). Prior to transformation of gonocytes into spermatogonia, an intermediate prespermatogonial stage occurred which had been ascertained as “quiescent” by Guraya [25]. The transformation of the gonocytes into spermatogonia was considered as initiation of spermatogenesis [26]. The first appearance of spermatogonia in the seminiferous tubules was an important factor as it denotes the onset of spermatogenesis. In the present study, the transformation of gonocytes to spermatogonia was observed in the seminiferous epithelium at 4 months of age (group-III) indicating the initiation of spermatogenesis at this age in Assam goat. However, Baishya et al. [27] recorded the initiation of spermatogenesis at 69 days of age in the Assam goats, which was much earlier as compared to 4 months of age (group-III) observed in this study. As the study was made at 2 and 4 months of age, the possibility of initiation of spermatogenesis after 2 months and prior to attaining 4 months of age could not be ascertained in the present work. The age of initiation of spermatogenesis was reported to be variable in different animals, which was recorded as 70 days in the Dwarf Nigerian lamb [21], 150 days in rams [6], 120 days in Brown Swiss crossbred bulls [28], and 350 days in buffalo [18].

**Figure 8:**
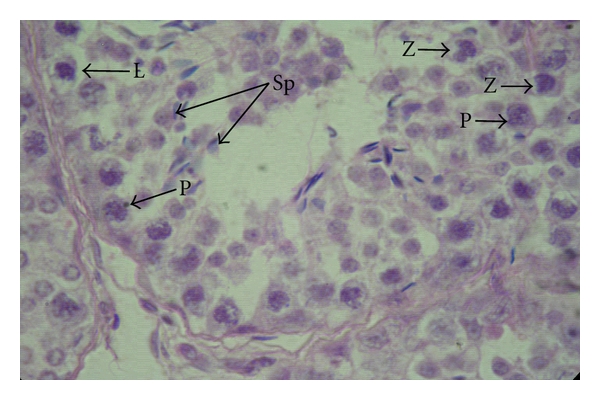
Photomicrograph showing leptotene (L), zygotene (Z), and pachytene (P) primary spermatocytes and spermatozoa (Sp) in the seminiferous epithelium of the testis of a six-month-old buck, H&E, 1000 X.

 The meiotic phases of the primary spermatocytes were recognized in the kids from 4 months of age (group-III) onwards. The leptotene primary spermatocytes were uniformly rounded cells with spherical nuclei. The chromatin substance was organized to form dense filamentous network which filled the nucleus completely. The cytoplasm was lightly stained. The nuclei were distinct spheroid shaped (Figure 8).

 The zygotene primary spermatocytes were marked by their deeply stained nuclei, which were usually notched, but occasionally rounded in shape. The chromatin substance appeared coarse. The nuclei were ill defined, and nuclear envelope was indistinct. The cytoplasm of the cells was pale stained (Figure 8).

 The pachytene primary spermatocytes were characterized by large spherical cells, and their nuclei were uniformly spheroid. The chromatin material appeared in coarse filamentous arrangement leaving irregular interstices in the nucleoplasm. The nuclear envelope was indistinct, and nucleoli were small and faintly stained. The cytoplasmic rim was comparatively broad and faintly stained (Figure 8).

**Figure 9:**
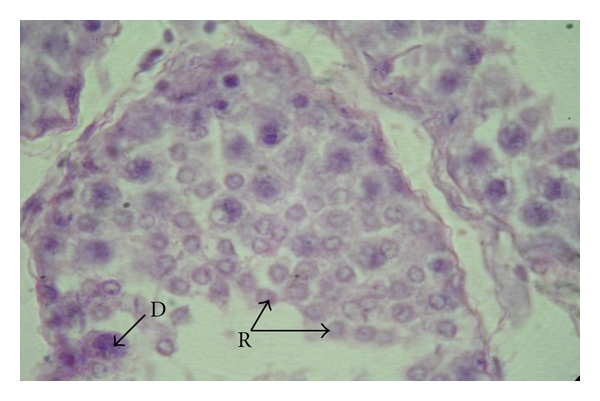
Photomicrograph of the testis in a six-month-old buck showing diplotene (D) primary spermatocytes and round spermatids (R) in the seminiferous epithelium, H&E, 1000 X.

 The diplotene primary spermatocytes were characterized by their largest size of the spermatogenic series. They were large, rounded cells with spherical nuclei. The chromatin material was more loosely arranged. They were stained lightly than the pachytene nuclei. The nuclear membrane was indistinct, and the nucleoli were rarely perceptible. The cytoplasmic rim surrounding the nucleus was weakly eosinophilic (Figure 9).

 The secondary spermatocytes were smaller in size than the primary spermatocytes but larger than the round spermatids. They were rounded in shape, and their nuclei were spherical with centrally placed small dot-like clumps of chromatin interconnected with fine filamentous network. The nuclear membrane was distinct with indistinct nucleoli. The cytoplasm was scant and eosinophilic. These cells were seen in the goats from 6 months of age (group-IV) onwards.

 The round spermatids (Figure 9) were the smallest cells of the spermatogenic series. They possessed spherical nuclei and had thin peripheral cytoplasmic rim. The chromatin substance appeared granular with 2 to 3 larger irregular aggregates which stained more intensely than the surrounding nuclear material. The nuclear membrane was thin and prominent.

 The elongated spermatids (Figure 7) were characterized by elongated nuclei with condensed chromatin. They stained more deeply and either remained attached to the Sertoli cell cytoplasm or migrated centripetally towards the lumina of the seminiferous tubules.

 The spermatozoa (Figure 8) were usually found attached to the Sertoli cells at their ad luminal border and in the lumen of the seminiferous tubules after their release from the Sertoli cells. The head was elongated—oval in shape. The present study confirmed the first occurrence of spermatozoa attached to the ad luminal border of the Sertoli cells or in the lumina of the seminiferous tubules at 6 months of age (group-IV), indicating the onset of puberty at this age in male Assam goats. The first occurrence of spermatozoa had been reported at 18 weeks of age in the Dwarf Nigerian ram [21], 165 days in native rams of Iran [29], and 20 weeks in Korean native goat [12]. The initiation of puberty as evidenced by presence of sperm cells in the ejaculated semen was found to be at 29 weeks in the Beetal X Assam local male kids [30].

 The histological characteristics of all the spermatogenic cells recorded in the present study in the Assam goats at various ages were in agreement with the earlier findings reported in the Dwarf Nigerian ram [21], domestic animals [31], goat [32], and native rams of Iran [29].

**Figure 10:**
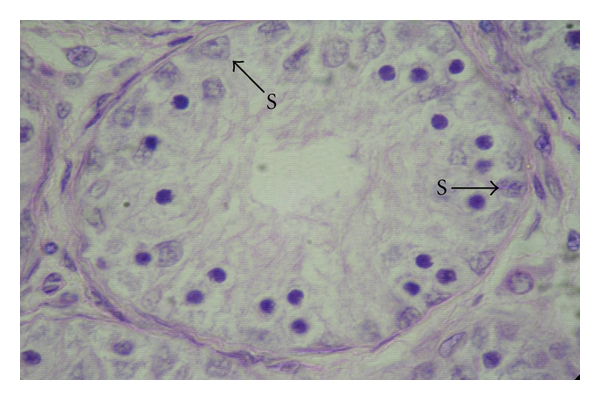
Photomicrograph of the testis in a four-month-old male kid showing Sertoli cell nuclei (S) in the seminiferous epithelium, H&E, 1000.

 The sustentacular cells (Sertoli cells) (Figure 10) were elongated pyramidal cells whose broad base rested on the basement membrane. The nuclei were spherical or pear-shaped and were located near the basement membrane of the seminiferous tubules. The centrally placed nucleolus was large and intensely stained. The nucleoli were absent in the nuclei of these cells at birth (group-I), which might be due to their immaturity. The nucleoli were present in the Sertoli cells from 2 month of age (group-II) onwards. The chromatin material of the nucleus was fine, evenly distributed, and stained lightly. The cellular outlines of the Sertoli cells were not distinct. As the process of spermatogenesis was fully established in the 6-month-old bucks (group-IV) onwards, morphological maturation of the Sertoli cells was noticed which included movement of nucleus to the basal portion of the cell, and nuclear membrane became folded with the development of prominent nucleolus and proliferation of organelles as evidenced from darkly stained masses in the basal region of the Sertoli cells as also reported in buffalo calves [10].
